# Estimation of Cutoff Values of Cotinine in Urine and Saliva for Pregnant Women in Poland

**DOI:** 10.1155/2013/386784

**Published:** 2013-10-21

**Authors:** Joanna Stragierowicz, Karolina Mikołajewska, Marta Zawadzka-Stolarz, Kinga Polańska, Danuta Ligocka

**Affiliations:** ^1^Department of Toxicology and Carcinogenesis, Nofer Institute of Occupational Medicine, 8 Teresy Street, 91-348 Lodz, Poland; ^2^Nofer Institute of Occupational Medicine, 8 Teresy Street, 91-348 Lodz, Poland; ^3^Department of Environmental Epidemiology, Nofer Institute of Occupational Medicine, 8 Teresy Street, 91-348 Lodz, Poland

## Abstract

Setting appropriate cutoff values and the use of a highly sensitive analytical method allow for correct classification of the smoking status. Urine-saliva pairs samples of pregnant women in the second and third trimester, and saliva only in the first trimester were collected. Offline SPE and LC-ESI-MS/MS method was developed in the broad concentration range (saliva 0.4–1000 ng/mL, urine 0.8–4000 ng/mL). The mean recoveries were 3.7 ± 7.6% for urine and 99.1 ± 2.6% for saliva. LOD for saliva was 0.12 ng/mL and for urine 0.05 ng/mL; LOQ was 0.4 ng/mL and 0.8 ng/mL, respectively. Intraday and interday precision equaled, respectively, 1.2% and 3.4% for urine, and 2.3% and 6.4% for saliva. There was a strong correlation between salivary cotinine and the uncorrected cotinine concentration in urine in the second and third trimesters of pregnancy. The cutoff values were established for saliva 12.9 ng/mL and urine 42.3 ng/mL or 53.1 **μ**g/g creatinine with the ROC curve analysis. The developed analytical method was successfully applied to quantify cotinine, and a significant correlation between the urinary and salivary cotinine levels was found. The presented cut-off values for salivary and urinary cotinine ensure a categorization of the smoking status among pregnant women that is more accurate than self-reporting.

## 1. Introduction

The most commonly used biomarker of exposure to tobacco smoke is cotinine, as a metabolite of nicotine. The measurement of the cotinine concentration in various biological fluids is directly proportional to the degree of exposure to nicotine [1]. The determination of cotinine is recommended for the assessment of active tobacco smoking, monitoring of environmental tobacco smoke (ETS) exposure, and impact evaluation of smoking cessation programs [2]. The most important advantage of using cotinine as a biomarker of tobacco smoke and ETS is the fact that about 72% of nicotine is converted to cotinine [3] and the half-life of cotinine averages about 17 hours, in comparison to the one averaging 2-3 h in case of nicotine [4]. The total nicotine content in tobacco (by weight of tobacco) averaged 10.2 mg [5], while the nicotine intake per cigarette averaged 1.04 mg [6], representing about 10%.

According to the Global Adult Tobacco Survey (GATS) in Poland, in 2009, approximately 24% of women aged 15–49 years were smokers (out of whom 21% were daily cigarette smokers) [7]. Based on the Pregnancy-related Assessment Monitoring Survey (PrAMS), the most recent results show that even more than 12% of pregnant women in Poland smoke [8].

The effect of tobacco smoking is not limited to the one of nicotine, which is responsible for addiction to smoking, but also involves the influence of various toxic substances released from burning cigarettes, like carbon monoxide, PAHs, heterocyclic compounds, N-nitrosoamines, aromatic amines, N-heterocyclic amines, aldehydes, or volatile hydrocarbons, among which 69 are known carcinogens [9–11]. The most important adverse health effects associated with maternal cigarette smoking are premature rupture of membranes, placental abruption or preeclampsia [12], uteroplacental insufficiency, and reducing the blood flow to the fetus. Maternal smoking may also result in lower birth weight of the newborns [11]. Based on the analysis performed in Poland, in the newborns prenatally exposed to ETS, the birth weight was lower by 335 g ± 90.3 than that in the case of the nonexposed newborns (*P* < 0.001) after adjustment for maternal educational level, marital status, prepregnancy weight, child gender, and gestational age [13]. Maternal nicotine exposure may cause changes in the development and maturing of the offspring's lungs, which can result in the organ being more susceptible to disease and likely to manifest reduced lung function [14]. Smoking during pregnancy may have long-term consequences on the neurobehavioral development of children [8].

Due to numerous highly adverse effects of smoking during pregnancy, there is a need to monitor the extent of exposure, spread the knowledge of these effects to the fetus, and promote smoking cessation. Evaluation of the smoking status among pregnant women is based mainly on a self-reported questionnaire. However, only a confirmation by a laboratory analysis may lead to correct and reliable classification since pregnant women (and not only they) are reluctant to admit that they smoke.

Interindividual variability in the metabolism of nicotine is due to the gender and ethnic differences in the activity of enzymes (CYP2A6 and UGT1A) and, to some extent, genetic polymorphisms of the *CYP2A6* gene [15]. Like many other physiological processes, also the metabolism of nicotine changes during pregnancy. The observed variability in the metabolic clearance of cotinine may markedly increase by 140% during pregnancy, resulting in a half-life shorter by nearly 50% than the one in the nonpregnant state [16].

The explanation of these changes could be the influence of a higher concentration of estradiol during pregnancy [17], which induces the activity of CYP2A6 responsible for the metabolism of nicotine [18]. 

As reported by Rebagliato et al. [19], the salivary cotinine level was significantly lower during pregnancy, compared with the postpartum one. Therefore, it is necessary to identify a cutoff value to avoid misclassification of smoking and nonsmoking pregnant women.

The ROC analysis is increasingly used to determine the cut-off values for biomarkers of exposure to tobacco smoke [20–23].

The primary aim of this study was to establish the optimal cut-off value for cotinine in saliva and urine of pregnant women in Poland and to compare the diagnostic effectiveness of three smoking tests: cotinine in saliva and in urine and in urine with creatinine correction. The secondary one was to develop a sensitive and specific method for determining the cotinine level in urine and saliva in a broad range of concentrations. Finally, our aim was also to estimate the utility of these matrices for both rapid screening used in order to identify potential smokers and more accurate determination of the degree of exposure to tobacco smoke, especially that concerning pregnant women.

## 2. Methods

### 2.1. Population

From the biobank of the Polish Mother and Child Cohort Study (REPRO PL), saliva-urine samples collected in trimester II and III of 69 women were selected as well as the survey data on the smoking status of the pregnant woman, the smoking habit of her husband/partner, and a consent to smoking in the apartment. In addition, each of these women had a saliva sample taken during the first trimester of pregnancy. The complete description of the cohort was published elsewhere [24]. In short, the inclusion criteria were single pregnancy up to 12 weeks of gestation, no assisted conception, no pregnancy complications, and no chronic diseases as specified in the study protocol [24]. The mean age of 69 women was 26.41 ± 4.97 years. Based on the survey data, it was found that in the first trimester 19/69 women were smokers and, in the second and third trimesters, this ratio equaled 17/69. Smoking was permitted in 52% of the apartments in the first trimester of pregnancy, but in the third trimester such permission to smoke at home decreased by approximately 9%. 

To find the correlations between the matrices, we analyzed saliva and urine samples collected at the same time.

### 2.2. Standards and Reagents

Cotinine (98%), internal standard-cotinine-d_3_ (98%), and ammonium acetate (98%) were obtained from Sigma Aldrich. Acetonitrile and Methanol Ultra Gradient HPLC Grade were supplied by Baker. Acetic acid (>99%) was purchased from Fluka. Ultrapure water was obtained from Milli-Q-Plus, Ultra-PureWater System (Millipore USA). All working standards of cotinine and cotinine-d_3_ were prepared in acetonitrile and stored at −20°C. Solid phase extraction manifold was maintained on Supelco, and OASIS HLB LP 96-Well Plate 60 *μ*m (60 mg) was supplied by Waters (USA). Control urine lyophilized ClinCheck/Control, for toxic organic compounds, was purchased from Recipe Chemicals (Germany).

### 2.3. Biological Samples Collection

Saliva was collected from pregnant women into a Salivette with citric acid (Sarstedt, Germany). The amount of approximately 1-2 mL of saliva was easily obtained by having the women chew a cotton swab, at least 30 min after eating or drinking. A clear, fluid sample was obtained by centrifuging the Salivette and used for analysis. A 50 mL volume of morning urine was collected from pregnant women to a 100 mL polypropylene container (Bene, Poland). All saliva and urine samples were transported to the laboratory in a cool box and stored at −20°C until analysis.

### 2.4. Samples Preparation

Urine and saliva samples were thawed before the analysis, thoroughly mixed, and transferred into 2.0 mL polypropylene tubes. The samples were centrifuged for 10 min at 11000 rcf (MIKRO 120, Hettich Zentrifugen, Tuttlingen, Germany). To 0.25 mL of urine or 0.5 mL of saliva, water and 20 *μ*L of internal standard (cotinine-d_3_) were added and mixed vigorously. Each well of the Oasis HLB extraction plate was prewashed with 2 mL of acetonitrile followed by 2 mL of water. Then, the samples were placed on the plate and washed with 1.0 mL of water (in case of saliva samples) and 1.0 mL of 20% methanol in water (in case of urine samples). The analytes were eluted with 1 mL of acetonitrile, and 20 *μ*L of the extract was injected into the chromatographic system. 

### 2.5. Calibration

The working solutions were prepared by appropriate dilutions of the standard stock solutions. The standard stock solutions of 1 mg cotinine or cotinine-d_3_/mL were further diluted with acetonitrile to obtain the working solutions of cotinine (10 *μ*g/mL, 400 ng/mL, and 20 ng/mL) and cotinine-d_3_ (2.5 *μ*g/mL). Calibration standards were prepared for saliva at the following concentrations: 0.2; 0.5; 1.0; 5.0; 10; 50; 100; 200; 500 ng/mL and for urine 0.2; 0.5; 1.0; 5.0; 10; 50; 100; 200; 500; 1000 ng/mL. All working solutions were stored at −20°C. Calibration curves were generated using linear regression with 1/X weighting.

### 2.6. Liquid Chromatography-Mass Spectrometry

The chromatographic separation was performed using the Waters 2695 Alliance LC System (Waters, USA) on the analytical column X-Terra MS C 18 3.5 *μ*m 2.1 × 150 mm (Waters). 

The following mobile phase was used: A 7% (water containing 0.04% of ammonium acetate with 0.05% of acetic acid) and B 93% (acetonitrile) with an isocratic mode and flow rate of 0.2 mL/min.

The Micromass Quattro Micro API tandem mass spectrometer (Waters, USA) was coupled to the HPLC Alliance system. The mass spectrometer was operated in the electrospray positive mode; the capillary was kept at 1.0 kV and the source temperature was maintained at 130°C, the desolvation gas flow was 600 L/h and the desolvation temperature was kept at 350°C, and the cone energy was 33 V and the collision energy was 21 eV for both cotinine and cotinine-d_3_. The specific ion transitions for cotinine and cotinine-d_3_ were monitored in a multiple reaction monitoring mode (MRM) with a dwell time of 0.3 s. 

### 2.7. Creatinine Correction

The creatinine level was determined according to the Jaffe automated method. Urine samples with a creatinine concentration lower than 0.3 g/L or higher than 3.0 g/L were excluded [25]. 

### 2.8. Statistical Analysis

Receiver operating characteristic (ROC) curve analysis was used for three purposes: to test the performance of the diagnostic system (how close to 1 is AUC), to compare the performance of the three tests, and to establish the optimal cut-off values for cotinine in saliva and in urine and creatinine-corrected concentration in urine. Statistical analysis was performed based on the measurement of cotinine in the biological material, taking into account the self-reported information on smoking and the data from the questionnaires concerning the ETS exposure at home of samples donors. The level of statistical significance was kept at *P* < 0.05. The ROC analysis module of the IBM program SPSS ver. 20.0 (IBM SPSS) was used for the ROC curves analysis and comparison of the area under the curve (AUC).

The correlation between the cotinine concentrations in saliva and urine was analyzed with the IBM SPSS Statistics 20.0.

## 3. Results

### 3.1. Optimization of the HPLC-MS/MS Conditions

The mass spectrometer conditions were optimized by monitoring cotinine and cotinine-d_3_ ion pairs for quantification in the MRM mode. The best results were achieved with m/z transitions of quantification traces, 177.2 > 80.2; 180.2 > 80.2, and confirmation, 177.2 > 98.2; 180.2 > 101.2, for cotinine and cotinine-d_3_, respectively.

The retention time of cotinine and cotinine-d_3_ was approximately 2.5 min, and the total run time equaled 6 min. The use of the structurally identical internal standard (cotinine-d_3_) eliminated most of the quantification errors.

### 3.2. Linearity

The relationship between the response and concentration of cotinine in a range of 0.4–1000 ng/mL (for saliva samples) and 0.8–4000 ng/mL (for urine samples) was linear, with the correlation coefficient (*r*) of the calibration curve *r* > 0.998 or higher. Detailed parameters of the method validation are presented in Table 1.

### 3.3. Sensitivity

The limit of detection (LOD) defined as the concentration of an analyte that gives at least a 3 : 1, signal to-noise ratio was 0.12 ng/mL (for saliva) and 0.05 ng/mL (for urine), and the limit of quantification (LOQ), defined as the concentration of an analyte that gives at least a 10 : 1 signal-to-noise ratio, was 0.4 ng/mL (for saliva) and 0.8 ng/mL (for urine).

### 3.4. Precision and Recovery

The intraday and interday precisions of the method were estimated by the analysis of control urine (ClinCheck, Level 1) and saliva samples (saliva from smokers) at the concentration of 248 ng/mL of urine (control range 198–298 ng/mL) and about 250 ng/mL of saliva on the same day and on four consecutive days (Table 2).

To determine the recovery of the extraction from the urine and saliva samples, seven different cotinine levels were added. Three repetitions of each concentration level were analyzed, and the ratio of the measured amounts to the added amounts was calculated (Table 3).

### 3.5. Tests of Usability: Smoking and Nonsmoking Women

To test the usability of the method, in total 138 pairs of urine-saliva samples were analyzed (69 pairs in trimester II and 69 pairs in trimester III), as well as 69 saliva samples collected in trimester I. In all urine and saliva samples, the concentrations of cotinine were above LOD (Table 4).

**Figure 1:**
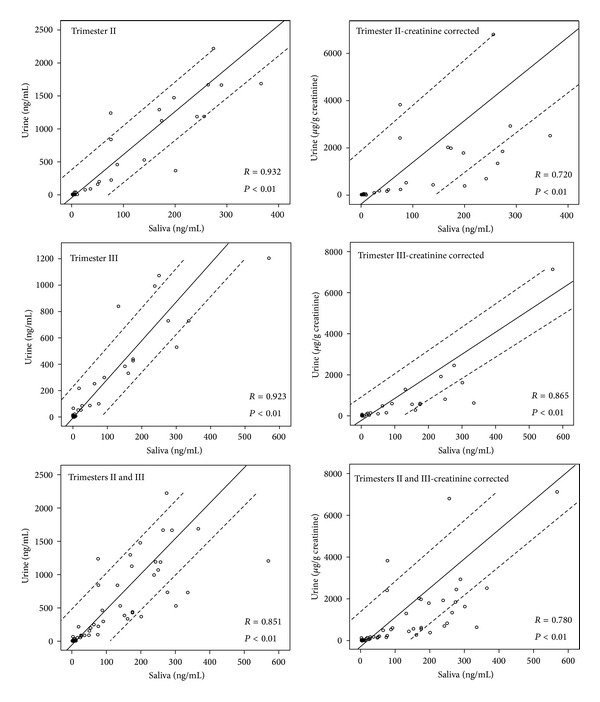
Comparison of the urinary and salivary cotinine concentrations, depending on the trimester of pregnancy and creatinine corrected concentration.

To assess the correlations between the matrices, the linear regression and Pearson's correlations were calculated. The results are shown in Figure 1. The correlations between the saliva and the uncorrected cotinine concentration in urine in trimester II (*r* = 0.932) and III (*r* = 0.925) were higher than the aggregated data of trimester II and III (*r* = 0.851), both being statistically significant (*P* < 0.01). Similar trends were observed for the correlation between saliva and creatinine-corrected cotinine in urine, but the corresponding *r* values were lower, that is, 0.720, 0.865, and 0.780, for trimester II, III, and the total period of pregnancy, respectively. 

To verify the smoking status, various questionnaires are usually taken into account. In this study, we took into account information on the smoking status obtained in the survey.

The research model was used to compare different matrices (saliva, urine) and the creatinine correction of cotinine concentrations of urine. Also, an analysis was conducted of the effect of the period in the pregnancy on the cut-off value. Statistical analysis was performed to compare the cotinine concentrations in the samples taken from women at different periods of pregnancy. Saliva samples were collected during periodic medical examinations in trimester I, II, and III of pregnancy and urine samples in trimester II and III.

Receiver operating characteristic (ROC) curve analysis is a graphical and quantitative technique used for determination of the optimal cut-off value. Selecting the optimal cut-off value for individual biomarkers of smoking was conducted using the Youden's index, J, which is defined as the maximum sum of sensitivity and specificity decreased by 1, that is: *J*_max⁡_ = (sensitivity + specificity) − 1 [26]. The cut-off value is “optimal” when the index has the maximum value.

On the basis of the content of cotinine in saliva, the cut-off values for the corresponding uncorrected cotinine in urine and creatinine-corrected urine were calculated. The optimized cut-off values and parameters of ROC curves analysis of cotinine in saliva and urine for pregnant women are shown in Table 5. 

When choosing the optimal cut-off value, it is important to take into account both sensitivity and specificity, and the maximum value of Youden's index was the decisive factor.

ROC analysis showed that the optimal cut-off value separating smokers and nonsmokers for saliva varies depending on the period of pregnancy and equals 31.9 ng/mL for the first trimester, 18.1 ng/mL for the second one, and 11.47 ng/mL for the last one with the sensitivity of 100% and specificity of 96% for all tests. In addition, the optimum cut-off value was determined on the level of 18.8 ng/mL (98.3%, 89.6, *n* = 241) for cotinine in saliva throughout pregnancy (trimesters I, II, and III).

The optimal cut-off value for cotinine in the urine samples collected in trimester II was higher than in trimester III both for the urine-53 ng/mL versus 34.44 ng/mL and the creatinine corrected concentration-68.35 *μ*g/g creatinine versus 48.53 *μ*g/g creatinine (Table 5).

The ROC analysis for the combined samples taken during the period from the second to the third trimester of pregnancy showed lower cut-off values for the cotinine level in all tested matrices, 12.45 ng/mL for saliva and 42.3 ng/mL and 53.09 *μ*g/g creatinine for urine with the sensitivity equaling 100% and specificity of 95%. (Table 5). 

The determined cut-off for cotinine in saliva samples collected at the beginning of the pregnancy was 31.9 ng/mL (sensitivity 100%/specificity 96%).

These values were established to distinguish between pregnant smokers and nonsmokers.

## 4. Discussion

We developed and validated the method for rapid, sensitive, and specific determination of cotinine in urine and saliva. By achieving the low limit of quantification (0.4 ng/mL for saliva and 0.8 ng/mL for urine), the presented method can be useful to assess the exposure to tobacco smoke as ETS and active smoking. Using the noninvasive methods of sampling biological material, like urine or saliva, the smoking status can be more easily assessed, especially with lower volume of biological fluids, than in other studies.

A number of other analytical methods have been applied for the measurement of cotinine in urine or saliva including immunological methods, for example, enzyme linked immunosorbent assay (ELISA, LOD = 1.3 ng/mL). However, cross-reactivity is still the major concern for immunological methods, especially with nicotine metabolites: 3′-hydroxycotinine and 3′-hydroxycotinine-glucuronide [27]. The most commonly used analytical techniques are gas chromatography with flame-ionization detection (GC-FID, LOQ = 500 ng/mL) [28], mass spectrometry (GC-MS, LOQ = 10 ng/mL) [29], and liquid chromatography with mass spectrometry (UPLC-MS/MS, LOQ = 1.1 ng/mL) [30, 31]. These methods have been used to distinguish smokers from nonsmokers. Many gas chromatographic methods are characterized by relatively high limits of quantification, which makes it more complicated to access the ETS exposure. In this case, the method of choice should make it possible to achieve a limit of detection lower than 1 ng/mL [32]. In order to evaluate such low concentrations, it is very important to minimize ion suppression, in particular in the chromatography method coupled with mass spectrometry or tandem mass spectrometry. Many analytical methods do not use internal standards or use structurally different internal standards than radiolabeled compounds, such as milrinone [33], diphenylamine [34], and acetaminophen [35], which may contribute to the increase of ion suppression [36].

A current state-of-the-art method for determination of cotinine in biological material is liquid chromatography with mass spectrometry (LC-MS/MS) and atmospheric pressure or electrospray ionization which gives high sensitivity. The results of our study offered a method characterized by improved sensitivity, selectivity, and high throughput in comparison to other conventional techniques. The structurally identical deuterated internal standard (cotinine-d_3_) increases the selectivity of the method, eliminates the effect of ion suppression, and results in higher precision and accuracy of the measurement. The advantage of the use of the 96-well extraction plate is the possibility to cleanup multiple samples, blank, and quality control samples, in parallel under the same conditions. This sample preparation procedure makes it possible to achieve a lower limit of detection (LOD = 0.05 ng/mL) by minimizing the matrix effect with only 0.25 mL of urine or 0.5 mL of saliva needed for the analysis.

So far, many studies have been published concerning cotinine cutoff points, yet only a few included pregnant women. In 1998, Klebanoff et al. [37] established a serum cotinine cut-off value 10 ng/mL for pregnant women which corresponds to the concentration in saliva of 12.5 ng/mL. This estimation is based on the study of Jarvis who discovered that the concentration of cotinine in saliva is 1.25 times higher than in serum [38]. For the nonpregnant women-14 ng/mL serum [39] and 12 ng/mL [40]. In Japan, for a large validation study (*n* = 5128) on tobacco smoke exposure during pregnancy, the cutoff for serum cotinine was established at 11.48 ng/mL which corresponds to 14.35 ng/mL in saliva [41]. As for saliva, the values obtained for pregnant women were on the level of 13 ng/mL [42] and 24 ng/mL [43, 44].

Interindividual variabilities in the metabolism of nicotine are due to the gender- and ethnicity-dependent differences in the activity of enzymes (CYP2A6 and UGT1A) or, to some extent, genetic polymorphisms of the *CYP2A6* gene [15]. 

As for urine, the results recorded among pregnant women were 100 ng/mL [45] and among the nonpregnant ones-50 ng/mL [39], 200 ng/mL [46], and 550 ng/mL [21].

From the physiological point of view, pregnancy is a dynamic process, during which there increases, inter alia, the volume of blood (30–40%) [47], distribution volume and rate of nicotine metabolism [16].

Probably, these are the main reasons for which the cut-off values for cotinine in saliva and urine at different stages of pregnancy may differ materially from those set for the general population. In our study, we compared changes in the concentration of cotinine in the samples taken from women at different times of pregnancy during periodic medical examinations. The cut-off values determined for trimester II, and III for both saliva and urine indicate a slight change (reduction). However, the difference between the values determined for the first trimester for saliva, that is, 31.9 ng/mL, was significantly higher for those determined for the second trimester and third trimester, respectively, 18.1 ng/mL and 11.47 ng/mL. In view of the relatively small changes in the metabolism of nicotine in early pregnancy, the cut-off value of 31.9 ng/mL for that period may be taken as a defining value for nonpregnant women.

The correct determination of the smoking status is particularly important in epidemiological studies assessing the effects of exposure to environmental factors on the outcome of pregnancy, where smoking is an important confounding factor that should be taken into account in the analysis and interpretation of results.

Minimizing the number of misclassified cases, we estimated the optimal cutoff point for pregnant women as 12.85 ng/mL for salivary cotinine and 42.3 ng/mL or 53.1 *μ*g/g creatinine for urinary cotinine. The observed slight decrease of cotinine cut-off values both for saliva and urine in trimester III as compared with II confirms the previous findings. These findings correspond to those obtained in a Nakayima and Yokoi's study where a large interindividual variability in cotinine N-glucuronidation (ca. 89-fold) in human microsomes in vitro was reported [14]. The hypothesis concerning a decrease of the free cotinine concentration in the body fluids of pregnant women may clarify our observation but still needs verification in the further research. 

In our study, cotinine concentrations in urine samples were compared with those in saliva samples to estimate the differences and correlations between the matrices. For the urinary and salivary cotinine levels in the second and third trimesters, high correlations were observed, better for saliva-urine than for saliva-creatinine-corrected urine. That confirms the findings of a previous study on the lesser use of creatinine correction [48, 49]. The mean urinary creatinine concentration in our study was 0.89 g/L (with the results <0.3 g/L, the mean was 0.82 g/L), which is a much lower result compared with the nonpregnant women aged 20–49, with the mean being 1.2 g/L [50]. A more reliable parameter for the assessment of diuresis would be obtained if the collection of 24-hour urine samples was used. However, in epidemiologic studies, spot samples are more practical than 24 hr urine samples [50]. 

For the monitoring of exposure to tobacco smoke, usually only one type of biological samples is collected. The choice of the sample type depends on the purpose of the study and the analytical technique that is available in the laboratory. This is particularly important in case of nonsmokers, where the expected concentrations are very low. Our study shows that the levels of cotinine in urine are about 4–5.5 times higher than in saliva, depending on the use of creatinine correction. On the other hand, saliva is a matrix that is easier to clean up than urine. All the advantages and disadvantages of those matrices must be taken into account while selecting the method of sample preparation and analytical technique. In this study, we used saliva samples for rapid screening of the smoking status. Urinary cotinine can be used to estimate more precisely the level of exposure to tobacco smoke, especially in nonsmokers, and to differentiate the nonexposed nonsmokers and exposed nonsmokers. In our analysis, we took into account only the survey data as a criterion for classification as smokers-nonsmokers, but a widespread lack of acceptance for smoking during pregnancy may be a cause of untrue answers.

In 2007, Zielińska-Danch et al. set a cut-off value for cotinine in urine of 500 ng/mL for the general population [21]. In later studies, Zielińska-Danch et al. [22] using ROC curve analysis obtained the value of 327 micrograms/g creatinine.

Exposure to the second-hand smoke is region specific, and in some countries a low level of exposure results in a much lower cutoff cotinine value. Until 2009, many studies referred to the cut-off value established by Jarvis et al. [39] that equaled 14 ng/mL serum for the general population, while the new value of 4.47 ng/mL (corresponding to 5.59 ng/mL saliva) for women was established as a result of the NHANES survey [20]. However, the authors proposed a general cut-off point of 3 ng/mL or ethnic-specific values between 1 and 6 ng/mL serum [20]. Benowitz et al. [20] established also a urine cut-off value of 15 ng/mL using an extrapolation of the above results and average ratios of cotinine in urine and plasma.

The cut-off value for cotinine in urine (determined by Goniewicz et al. [23]) equaling 31.5 ng/mL (*n* = 637) represents the multiethnic population, including the Poles, that is likely to be exposed to tobacco smoke in the same way as the group of pregnant women studied in our research.

The optimal saliva cut-off value of 12.9 ng/mL is comparable to that equaling 14.35 ng/mL saliva (derived form 11.48 ng/mL plasma) obtained for Japanese pregnant women [41]. Most likely, the high cutoff point concerning Polish pregnant women represents a similar second-hand exposure to tobacco smoke. Taking into account the smoke-free law, the substantial reduction of the ETS exposure can be expected, as well as a decrease of tobacco smoke biomarkers (cotinine in saliva and urine). As a consequence, also lower a cut-off value might be established.

## 5. Conclusion

The results of the analytical method validation indicate that the developed procedure can be applied for routine determination of cotinine in urine and saliva samples. Achieving a low limit of quantification (0.4 ng/mL for saliva and 0.8 ng/mL for urine) allows not only distinguishing between smokers and nonsmokers but also quantifing the exposure to environmental tobacco smoke and degree of active smoking. Our results showed a significant correlation between the urinary and salivary cotinine levels. Our study presents for the first time results of ROC curve analysis used to determine the cut-off values for the assay of cotinine in saliva and urine and in creatinine-corrected urine as a marker of exposure to tobacco smoke in women during different periods of pregnancy in order to distinguish their status of smoking. This is the first such study of pregnant women in Poland.

The ROC analysis with the application of the Youden's index helped to determine the optimal cut-off value for cotinine in saliva (18.9 ng/mL) and in urine (42.3 ng/mL and 53.1 *μ*g/g creatinine) for the first time for pregnant women in Poland.

The analysis of the diagnostic usefulness of cotinine determination in saliva and urine and the evaluation of the usefulness of the creatinine correction of the cotinine level showed that all three proposed cut-off values are characterized by high sensitivity and specificity.

Our results suggest that, during the interpretation of the analysis of cotinine, the period of pregnancy when the samples of urine or saliva are taken for the assessment of exposure to tobacco smoke may have some significance.

## Figures and Tables

**Figure 2:**
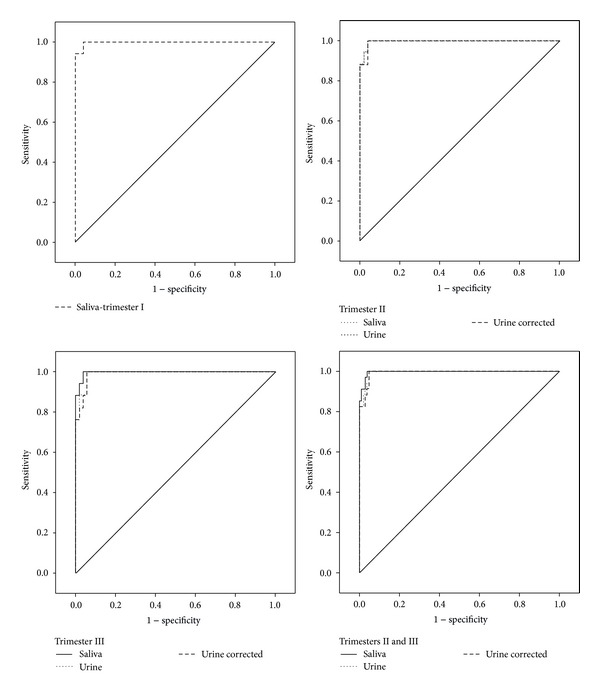
ROC curves analysis of smoking status based on questionnaires and measurement of cotinine in saliva and in urine and creatinine corrected cotinine in urine are shown in Figure 2.

**Table 1:** Validation parameters of the LC-MS/MS method of determination of cotinine in saliva and urine.

	LOD [ng/mL]	LOQ [ng/mL]	Range of linearity [ng/mL]	Correlation coefficient *r* ^2^	Uncertainty (*k* = 2) [%]	Accuracy [%]	Recovery* [%]
Urine	0.05	0.8	0.8–4000	0.9997	9.3	4.27	93.72 ± 7.6
Saliva	0.05	0.4	0.4–1000	0.9993	6.0	2.33	99.10 ± 2.6

*All values are means ± SD.

**Table 2:** Recovery of cotinine in urine and saliva samples.

	Expected value [ng/mL]	Measured* [ng/mL]	Recovery [%]
Urine Before spiking 0.884 ± 0.055 ng/mL	0.8	0.63 ± 0.01	78.5
	40	38.8 ± 3.4	97
	80	76.4 ± 3	95.6
	120	113.1 ± 4.6	94.3
	2000	1977 ± 96.3	98.8
	3800	3728 ± 76.6	98.1

Saliva Before spiking 0.701 ± 0.041 ng/mL	0.4	0.40 ± 0.037	100.5
	20	20.06 ± 0.56	100.3
	40	38.4 ± 4.2	95.9
	80	80 ± 2.9	100.5
	120	113.6 ± 2.2	94.7
	500	506.1 ± 7.36	101.1
	900	907 ± 10.5	100.7

*All values are means ± SD.

**Table 3:** Intraday and interday precision of cotinine in urine and saliva samples.

Sample	Intraday precision			Interday precision		
	*n*	Mean [ng/mL]	RSD [%]	*n*	Mean [ng/mL]	RSD [%]
Urine	3	257.5	1.2	12	259.0	3.4
Saliva	4	243.3	2.3	16	244.9	6.4

**Table 4:** Cotinine in saliva and urine classification based on self-reporting smoking status.

		*N*	AM	SD	GM	95% CI
Nonsmoking						
Saliva	Trimester I	50	5.99	6.88	4.437	4.376–4.498
	Trimester II	52	4.86	8.42	3.190	3.116–3.263
	Trimester III	52	3.86	4.14	3.060	3.024–3.096
Urine	Trimester II	52	10.78	29.31	4.497	4.242–4.751
	Trimester III	52	10.27	32.50	3.070	2.787–3.352
Urine corrected	Trimester II	52	14.33	35.61	5.531	5.221–5.841
	Trimester III	52	14.43	30.14	5.546	5.267–5.826
Smoking						
Saliva	Trimester I	19	175.77	91.69	151.88	150.561–153.199
	Trimester II	17	174.42	98.67	141.92	140.424–143.425
	Trimester III	17	181.40	139.97	128.45	126.319–130.577
Urine	Trimester II	17	1021.97	632.79	747.67	738.044–757.292
	Trimester III	17	500.15	368.45	341.72	336.115–347.323
Urine corrected	Trimester II	17	1758.4	1703.8	1017.31	991.395–1043.22
	Trimester III	17	1133.2	1685.8	545.10	519.465–570.741

**Table 5:** Optimized cutoff values of cotinine in saliva and urine for pregnant women (Youden's index 0.956 ± 0.0084).

	Smoking		AUC*	SE	95% CI	Cutoff	Sensitivity	Specificity
	Yes	No						
Saliva						[ng/mL]		
Trimester I	19	50	0.998	0.003	0.992–1.000	31.90	100%	96%
Trimester II	17	52	0.997	0.004	0.989–1.000	18.10	100%	96%
Trimester III	17	52	0.997	0.004	0.989–1.000	11.47	100%	96%
Trimesters II & III	34	104	0.997	0.003	0.992–1.000	12.85	100%	96%
Urine						[ng/mL]		
Trimester II	17	52	0.997	0.004	0.989–1.000	53.00	100%	96%
Trimester III	17	52	0.991	0.008	0.976–1.000	34.44	100%	94%
Trimesters II & III	34	104	0.994	0.004	0.997–1.000	42.31	100%	95%
Urine corrected						[*μ*g/g]		
Trimester II	17	52	0.995	0.005	0.986–1.000	68.35	100%	96%
Trimester III	17	52	0.990	0.008	0.974–1.000	48.53	100%	94%
Trimesters II & III	34	104	0.993	0.004	0.984–1.000	53.09	100%	95%

*P < 0.0001.
